# Magnitude of unintended pregnancy and its determinants among childbearing age women in low and middle-income countries: evidence from 61 low and middle income countries

**DOI:** 10.3389/frph.2023.1113926

**Published:** 2023-07-17

**Authors:** Fantu Mamo Aragaw, Tsegaw Amare, Rediet Eristu Teklu, Biresaw Ayen Tegegne, Adugnaw Zeleke Alem

**Affiliations:** ^1^ Department of Epidemiology and Biostatistics, Institute of Public Health, College of Medicine and Health Sciences, University of Gondar, Gondar, Ethiopia; ^2^ Department of Health Systems and Policy, Institute of Public Health, College of Medicine and Health Sciences, University of Gondar, Gondar, Ethiopia; ^3^ Department of Anesthesia, College of Medicine and Health Sciences, University of Gondar, Gondar, Ethiopia

**Keywords:** unintended pregnancy, multilevel analysis, low and middle-income countries, child bearing age, women

## Abstract

**Background:**

Unintended pregnancy is one of the most serious health issues in low and Middle-Income Countries (LMICs), posing significant health, economic, and psychosocial costs to individuals and communities. However, there is limited evidence on the prevalence of unintended pregnancies and their determinants in LMICs. Hence, this study aimed to assess the prevalence of unintended pregnancy and its associated factors among childbearing-age women in LMICs.

**Method:**

Data for the study were drawn from a recent 61 Demographic and Health Surveys (DHS) conducted in LMICs. A total sample of 187,347 mothers who gave birth in the five years preceding the survey was included. STATA version 16 was used to clean and analyze the data. Multilevel multivariable logistic regression was employed to identify individual and community-level factors of unintended pregnancy in LMICs. In the multivariable analysis, an adjusted odds ratio with a 95% confidence level was reported to indicate statistical association.

**Results:**

The pooled magnitude of unintended pregnancy in LMICs was 26.46%% (95% CI: 25.30%, 27.62%), ranging from 19.25%% in Egypt to 61.71% in Bolivia. Working status (AOR =  1.03; 95% CI: 1.01, 1.06), having a husband with no education (AOR = 1.07; 95% CI: 1.00, 1.15), and primary education (AOR =  1.05; 95% CI: 1.01, 1.11), women from male-headed households (AOR = 1.04; 95% CI: 1.00, 1.08), media exposure (AOR = 1.05; 95% CI: 1.02, 1.08), unmet need for contraception (AOR = 1.05; 95% CI: 1.02, 1.08), distance from a health facility (AOR = 1.03; 95% CI: 1.00, 1.06) were significantly associated with unintended pregnancy.

**Conclusion:**

Unintended pregnancy rates remain high in LMICs. Women whose husband has no education and primary education, women with media exposure, working status, women who live in a household headed by male, women with unmet need for contraception, and women with a big problem of distance to health facilities were variables that were significant predictors of unintended pregnancy. When attempting to minimize unintended pregnancy in LMICs, these factors need to be considered. Furthermore, most of these attempts should be driven by government entities in low and middle-income countries.

## Background

Unintended pregnancies are pregnancies that are unwanted and/or mistimed at the time of conception (1, 2). Because of the consequences associated with both the mother's and child's social and health outcomes, unintended pregnancy is a major public health concern in both high-income countries and Middle-Income countries (LMICs) (3–5). It is estimated that 208 million pregnancies occur worldwide each year, with 46% of them becoming unintended (6). Annually, more than 14 million unintended pregnancies were reported in Sub-Saharan Africa (7). Although the global trend of unintended pregnancy has decreased over time, it remains high with significant regional variations (6, 8–11).

Although it is a problem in both high-income countries and LMICs, unintended pregnancy has decreased in high-income countries compared to LMICs (7). In some areas, unintended pregnancy accounts for more than half of all pregnancies (12). In low-income countries, the rate of unintended pregnancy varies between 7.2 and 59.6 per 100 person-years of follow-up (11).

Maternal and neonatal mortality remain unresolved public health problems (12–15). Unintended pregnancy causes maternal morbidity and mortality as a result of the complications of unsafe abortion, miscarriage, and unplanned births (16–18). Unintended pregnancy has serious consequences for women's and children's health and well-being (19). Abortions occur in more than half of unintended pregnancies (12, 20). Unintended pregnancies can lower the use of maternal (21–24), and neonatal (25) health services utilization, and worsen maternal health outcomes (2, 26–28). Moreover, unintended pregnancy is associated with malnutrition, mental illness, and vertical transmission of the Human immunodeficiency virus (HIV) to children (29, 30).

Maternal health issues are the first key research area in sexual and reproductive health till 2030 (31). Unintended pregnancy is one of the most serious public health problems that impose major health, economic, and psychosocial costs on individuals and communities, as well as significant emotional damage to women, families, and society (32–34). Besides, unintended pregnancy is associated with preeclampsia, obstetric bleeding, stigma, and socioeconomic inequalities (2, 27, 35, 36).

Different variables such as maternal age (37–39), marital status (37, 39, 40), wealth status (7, 38, 41), having an occupation (42), educational status of women (1, 19, 41–43), media exposure (37, 44), parity (37, 38, 45), family size (37, 45), contraceptive use (38, 42), being victims of sexual violence (46–48) were found predictors of unintended pregnancy.

Tackling unintended pregnancy is one way to reduce the high rates of maternal and neonatal mortality (20, 24–26, 49, 50). To the best of our knowledge, no studies have been conducted to investigate the magnitude of unintended pregnancy in LMICs. Because the majority of unintended pregnancies occur in low- and middle-income countries, there is a critical need to explore the underlying causes of unintended pregnancies among women in these countries. Hence, the primary goals of this study were to determine the prevalence of unintended pregnancy and to assess the effects of potential underlying factors on unintended pregnancy among women aged 15–49 in LMICs.

## Methods

### Study population and data sources

This study used the Demographic Health Survey (DHS) data from 61 low and middle-income countries collected between 2008 and 2020. By measuring key indicators deemed important, a DHS survey allows countries to generate data that can be used to inform policy and practice. Each country's survey includes a variety of datasets such as men, women, children, birth, and household datasets. For this study, we used individual record (IR) data. To select study participants, the DHS employs a two-stage stratified sampling technique.

This study only included women between the ages of 15 and 49 who had given birth within the previous five years of the survey. As a result, the total sample size was 187,347, with respondents from each country ranging from 607 in Guyana to 9,534 in Nigeria. A detailed description of the survey year, sample size, and sample characteristics is presented in Table 1.

**Table 1:** Sample size determination in the study of the magnitude of unintended pregnancy among pregnant women, LMICS, DHS, 2008–2020.

Country	Year of survey	Sample size	Country	Year of survey	Sample size
Afghanistan	2015	9,065	Liberia	2019/20	1,817
Albania	2017/18	995	Lesotho	2014	1,177
Armenia	2015/16	598	Madagascar	2008/2009	3,921
Angola	2015/16	3,953	Chad	2014/15	5,169
Bangladesh	2017/18	2,333	Mali	2018	3,055
Burkina Faso	2010	4,785	Myanmar	2015/16	1,691
Benin	2017/18	4,168	Maldives	2016/17	1,102
Bolivia	2008	2,669	Malawi	2015/16	6,190
Burundi	2016/17	4,147	Mozambique	2011	3,621
Central Democratic Congo	2013/14	5,041	Nigeria	2018	9,534
Congo	2011/12	2,583	Niger	2012	3,681
Cote more	2011/12	2,430	Namibia	2013	1,847
Cameroon	2018	3,010	Nepal	2016	1,917
Colombia	2015	3,445	Papua New Guinea	2016/18	3,008
Dominican Republic	2013	1,054	Philippines	2017	3,142
Egypt	2014	6,505	Pakistan	2017/18	3,109
Ethiopia	2016	3,567	Rwanda	2019/20	2,998
Gabon	2012	1,659	Sierra Leone	2019	3,340
Ghana	2014	1,855	Senegal	2010	3,454
The Gambia	2019/20	2,478	Sao tome & Principe	2008/2009	610
Guinea	2018	2,570	Togo	2013	2,281
Guatemala	2014/15	4,399	Tajikistan	2017	2,003
Haiti	2016/17	4,268	Timor Leste	2016	4,804
Guyana	2009	607	Turkey	2013	924
Honduras	2011/12	3,609	Tanzania	2015/16	3,277
Indonesia	2017	5,951	Ukraine	2007	514
Jordan	2017/18	3,175	Uganda	2016	4,631
Kenya	2014	5,772	South Africa	2016	1,443
Cambodia	2014	2,780	Zambia	2018	3,406
Comoros	2012	972	Zimbabwe	2015	2,366
Kyrgyz Republic	2012	1,401			

### Study variables and measurements

The outcome variable was unintended pregnancy, which is composed of both pregnancies that are wanted no more or wanted later (mistimed). It was a binary variable, women with mistimed pregnancies or unwanted pregnancies were recorded as “unintended pregnancies”, while those who needed pregnancy then were recorded as ‘intended pregnancy’ (13). The study included individual-level independent variables such as the age of women (15–19, 20–24, 25–29, 30–34, 35–39, 40–44, and 45–49 years), educational status of women (no education, primary, secondary, and higher), educational status of the husband (no education, primary, secondary, and higher), media exposure (yes or no), working status (working or not working), terminated pregnancy (yes or no), household wealth status (poorest, poorer, middle, richer, or richest), household members (≤5, 6–10 or >10), and sex of household head (male or female), intention to use contraceptive (yes or no), and unmet need for contraceptive (yes or no). Community-level factors such as place of residence (rural or urban) and distance to health facilities (big problem and not a big problem) were also included.

Media exposure was created by asking women about the frequency of radio, television, and newspapers. It is classified as “yes” if women had at least one type of media exposure, such as radio, newspaper, or television, and “no” otherwise.

### Data processing and analysis

We appended the data from 61 LMICs after extracting the variables based on existing literature. Before any statistical analysis, the data were weighted using sampling weight to restore the survey's representativeness. The data was cleaned and statistical analyses were carried out using STATA version 16. Frequencies and percentages were used to describe the background characteristics of the study participants. We conducted a multilevel logistic regression analysis, assuming that each community has a different intercept and fixed coefficient, with a random effect applied at the cluster level. Factors with a *p*-value ≤0.2 in crude odds ratio (COR) were selected as candidates for the adjusted model, finally, the adjusted odds Ratio (AOR) with 95% CI was reported, and variables with *p*-values less than 0.05 were declared to be significant predictors of unintended pregnancy in the multivariable analysis.

### Parameter estimation method and model building

The fixed effects method was utilized to estimate the relationship between unintended pregnancy and independent variables, which was expressed as an odds ratio with a 95% confidence interval and a *p*-value of 0.05.The random effects, which are measures of variation of unintended pregnancy across communities or clusters, were expressed in terms of the Intra-Class Correlation (ICC), the median odds ratio (MOR), and the proportional change in variance (PCV). The ICC shows the differences between clusters in unintended pregnancy among reproductive-aged women. The ICC is calculated as $\mathrm{ICC}=\frac{\mathrm{VA}}{\;\mathrm{VA}+3.29}\times 100$, Where; VA represents the area-level variance (51–53). The MOR indicates the central value of the odd ratio between the highest and the lowest risk regions when two clusters are chosen at random. The MOR is calculated as MOR=e0.95√VA, where VA donates the area level variance (54, 55).

The PCV measures the proportion of total observed individual variation that can be attributed to differences between clusters. The PCV is calculated as; $\frac{V_{\mathrm{null}}-\mathrm{VA}}{V_{\mathrm{null}}}\times 100$, whereas; *V*_null_ represents the variance of the initial model, while VA represents the variance of the model with more terms (54, 55).

Four models were fitted to select the best-fit model for the data using deviance: the null model (model without independent variables), model I (model with individual-level variables), model II (models including community-level variables), and model III (model with both individual and community-level variables). Deviance information criteria (DIC) (−2 × log-likelihood value) were used to assess the goodness of fit. The Variance Inflation Factor (VIF) was used to test for multicollinearity among the selected independent variables.

### Ethical considerations

The data set was obtained from the DHS website after a formal request and permission from the major DHS. All methods were performed following the Demographic and Health Surveys (DHS) program's relevant guidelines and regulations. The dataset was not allowed to be shared with other organizations and has remained confidential.

## Results

### Background characteristics of study participants

Majority of the participants are in the age group of 25–29 [49,946(26.66%)]. Single women had a greater proportion of unintended pregnancies (27.75%), while the least proportion was recorded among married women (25.90%). Women who are from urban areas had a greater proportion of unintended pregnancies (26.57%) than women from rural areas (25.80%). Women who are from the poorest households have a higher proportion of unintended pregnancies (26.65%) compared to women from the richest household (25.80%) (Table 2).

**Table 2:** Relationship between individual and community level variables and unintended pregnancy among pregnant women, LMICS, DHS, 2008–2020.

Variables	Categories	Unintended pregnancy		Total weighted frequency (%)
		Yes	No (%)	
Age of women	15–19	3,282 (26.09)	9,295 (73.91)	12,577 (6.71)
	20–24	11,079 (26.16)	31,264 (73.84)	42,343 (22.60)
	25–29	13,082 (26.19)	36,863 (73.81)	49,946 (26.66)
	30–34	10,218 (26.19)	28,804 (73.81)	39,022 (20.83)
	35–39	6,950 (25.74)	20,055 (74.26)	27,005 (14.41)
	40–44	3,215 (25.69)	9,300 (74.31)	12,515 (6.68)
	45–49	1,031 (26.19)	2,906 (73.81)	3,937 (2.10)
Women education status	No education	14,657 (25.04)	43,867 (74.96)	58,525 (31.24)
	Primary	15,351 (26.81)	41,918 (73.19)	57,269 (30.57)
	Secondary	14,876 (26.17)	41,959 (73.83)	56,835 (30.34)
	Higher	3,974 (27.00)	10,744 (73.00)	14,718 (7.86)
Husband education status	No education	12,277 (25.24)	36,358 (74.76)	48,635 (29.44)
	Primary	12,097 (26.36)	33,796 (73.64)	45,894 (27.78)
	Secondary	14,006 (25.79)	40,304 (74.21)	54,311 (32.88)
	Higher	4,144 (25.36)	12,200 (74.64)	16,344 (9.89)
Marital status	Single	2,859 (27.75)	7,446 (72.25)	10,306 (5.50)
	Married	42,654 (25.90)	122,036( 74.10)	164,690( 87.91)
	Separated/divorced/widowed	3,342 (27.07)	9,006 (72.93)	12,349 (6.59 )
Wealth index	Poorest	10,730 (26.65)	29,052 (74.07)	40,261 (21.49)
	Poorer	10,171 (25.93)	28,435 (74.36)	39,224 (20.94)
	Middle	9,806 (25.64)	27,226 (73.83)	38,241 (20.41)
	Richer	9,652 (26.17)	27,226 (73.83)	36,878 (19.68)
	Richest	8,497 (25.95)	24,243 (74.05)	32,740 (17.48)
Occupation of women	Not working	22,729 (25.73)	65,611 (74.27)	88,341 (48.07)
	Working	25,161 (26.36)	70,286 (73.64)	95,448 (51.93)
Household size	1–5	22,609 (26.26)	63,479 (73.74)	86,088 (45.95)
	6–10	20,661 (26.01)	58,766 (73.99)	79,428 (42.40)
	>10	5,586 (25.59)	16,243 (74.41)	21,830 (11.65)
Sex of household head	Male	39,548 (26.05)	112,262 (73.95)	151,811 (81.03)
	Female	9,309 (26.20)	26,226 (73.80)	35,536 (18.97)
Media exposure	No	12,727 (25.26)	37,649 (74.74)	50,375 (26.89)
	Yes	36,130 (26.38)	100,840 (73.62)	136,970 (73.11)
Unmet need for contraception	Yes	11,403 (26.33)	31,907 (73.67)	43,310 (23.12)
	No	37,454 (74.00)	106,582 (26.00)	144,036 (76.88)
Intention to use contraception	Yes	14,812 (26.26)	41,597 (73.74)	56,410 (50.16)
	No	14,176 (25.29)	41,881 (74.71)	56,057 (49.84)
Community level variables				
Residence	Urban	17,784 (26.57)	491,445 (73.43)	66,929 (35.72)
	Rural	31,073 (25.80)	89,344 (74.20)	120,417 (64.28)
Distance to the health facility	Big problem	17,939 (25.99)	51,095 (74.01)	69,034 (36.85)
	Not big problem	30,918 (26.13)	87,393 (73.87)	118,313 (63.15)

### The pooled prevalence of unintended pregnancies

**Figure 1:**
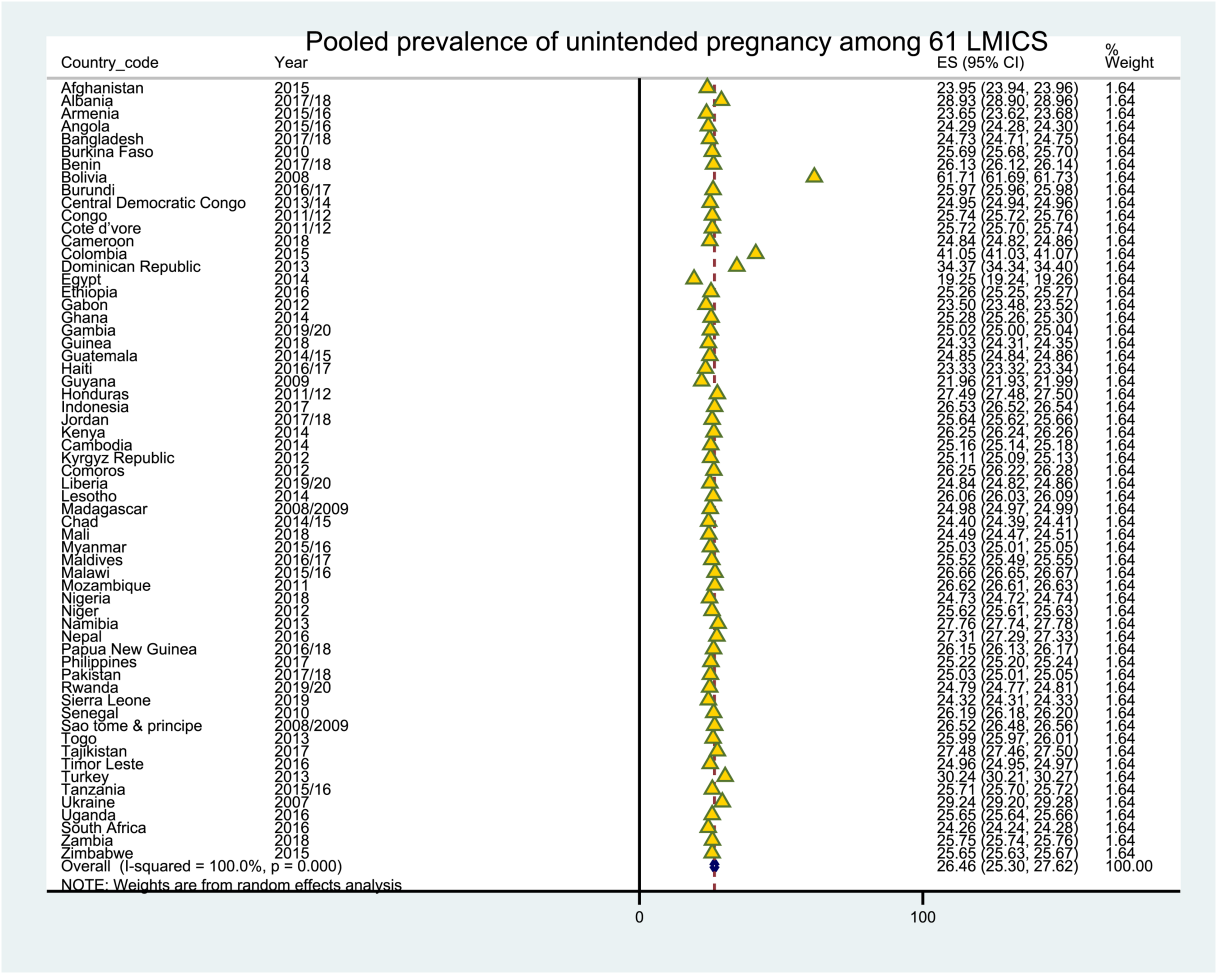
The pooled prevalence of unintended pregnancy among 61 LMICS.

The overall prevalence rate of unintended pregnancy in LMICs was 26.08% (95% CI: 26.00%, 26.41%). The highest prevalence of unintended pregnancy was reported in Bolivia at 61.71% (95% CI: 61.69%, 61.73%), and the lowest proportion of unintended pregnancy was recorded in Egypt at 19.25% (95% CI: 19.24%, 19.26%) (Figure 1).

### Factors associated with unintended pregnancy

Based on the final model (Model III, which includes both individual and community-level variables), occupation, husband's education, unmet need for contraceptive, and media exposure, and distance to health facilities were variables statistically associated with unintended pregnancy.

Women whose husbands have no education and primary education were 1.07 (AOR = 1.07; 95% CI: 1.00, 1.15), and 1.09 (AOR = 1.09; 95% CI: 1.02, 1.17) times higher odds of experiencing unintended pregnancies respectively than women who have higher education. The odds of unintended pregnancy in those who are currently working are 1.03 (AOR =  1.03; 95% CI: 1.00, 1.06) times higher than that of women who are not working. The risk of unintended pregnancy among women who live in a household with a male head was 1.04 (AOR = 1.04; 95% CI: 1.00, 1.08) times higher as compared to women who live in a household with a female head. Women who are exposed to media have 1.05 (AOR = 1.05; 95% CI: 1.02, 1.08) times higher odds of having an unintended pregnancy than those women who have not been exposed to media. The odds of experiencing unintended pregnancy among those women who had an unmet need for contraception were 1.05 (AOR = 1.05; 95% CI: 1.02, 1.08) times higher compared to those without an unmet need for contraception. Mothers who perceive distance from the health facility as a big problem had 1.03 (AOR = 1.03; 95% CI: 1.00, 1.06) times higher odds of having an unintended pregnancy compared to their counterparts (Table 3).

**Table 3:** Multilevel multivariable analysis of factors associated with unintended pregnancy among women in LMICs, DHS 2008–2020.

Variables	categories	Null model	Model I	Model II	Model III
			AOR [95% CI]	AOR [95% CI]	AOR [95% CI]
Age of women	15–19		1.03 [0.97, 1.19]		1.03 [0.97, 1.11]
	20–24		0.99 [0.94, 1.04]		0.99 [0.94, 1.04]
	25–29		1.00 [0.96, 1.05]		1.00 [0.96, 1.05]
	30–34		0.99 [0.95, 1.04]		0.99 [0.95, 1.04]
	35–39		1.00		1.00
	40–44		0.98 [0.91, 1.04]		0.98 [0.91, 1.04]
	45–49		0.99 [0.90, 1.08]		0.99 [0.90, 1.09]
Women education status	No education		0.99 [0.91, 1.08]		0.99 [0.91, 1.07]
	Primary		1.05 [0.96, 1.14]		1.04 [0.96, 1.13]
	Secondary		1.02 [0.95, 1.12]		1.02 [0.94, 1.12]
	Higher		1.00		1.00
Husband education status	No education		1.07 [1.00, 1.15]*		1.07 [1.00, 1.15]*
	Primary		1.09 [1.02, 1.17]*		1.09 [1.02, 1.17]*
	Secondary		1.03 [0.97, 1.10]		1.03 [0.97, 1.10]
	Higher		1.00		1.00
Occupation of women	Not working		1.00		1.00
	Working		1.03 [1.00, 1.06]**		1.03 [1.00, 1.06]**
Household size	1–5		1.01 [0.97, 1.06]		1.01 [0.97, 1.06]
	6–10		0.99 [0.95, 1.03]		0.99 [0.95, 1.03]
	>10		1.00		1.00
Terminated pregnancy	No		1.00		1.00
	Yes		1.00 [0.96, 1.04]		1.00[0.96,1.04]
Sex of household head	Male		1.04 [1.00, 1.08]*		1.04 [1.00, 1.08]*
	Female		1.00		1.00
Wealth index	Poorest		1.00		1.00
	Poorer		0.99 [0.95, 1.03]		0.99 [0.95,1.03]
	Middle		1.00 [0.96,1.05]		1.00 [0.96, 1.05]
	Richer		1.00 [0.96,1.05]		1.00 [0.96, 1.05]
	Richest		0.96 [0.92, 1.02]		0.96 [0.91, 1.02]
Knowledge of the ovulatory cycle	Yes		1.00		1.00
Media exposure	No		1.00		1.00
	Yes		1.05 [1.01, 1.08]*		1.05 [1.01, 1.08]***
	No		0.98 [0.95, 1.02]		0.98 [0.95, 1.02]
Intention to use contraceptive	Yes		1.00		1.00
	No		0.98 [0.96, 1.01]		0.95 [0.98, 1.01]
Unmet need for contraception	Yes		1.00		1.00
	No		1.05 [1.02, 1.08]*		1.05 [1.02, 1.08]***
Community level variables					
Residence	Rural			1.00	1.00
	Urban			1.04 [1.01, 1.06]*	1.01 [0.97, 1.05]
Distance to the health facility	Not big problem			1.00	1.00
	Big problem			1.01 [0.99, 1.03]	1.03 [1.00, 1.06]***
Random effect					
	VA	0.072	0.012	0.07	0. 012
	ICC	0.021	0.003	0.02	0.003
	MOR	0.69	0.28	0.68	0.28
	PCV (%)	Reference	83%	2%	83%
Model comparison					
	Deviance	218,505	116,433	218,492	116,427

AOR, adjusted odds ratio; CI, confidence interval; ICC, inter cluster correlation coefficient; MOR, median odds ratio; PCV, proportional change in variance; VA, area level variance.

* *p* value <0.05.

** *p* value <0.01.

*** *p* value <0.001.

### Random effects model and model fitness

The random effects results are shown in Table 3. It was found that in the empty model (Model 0), there are substantial variations in unintended pregnancies among LMICs. The ICC in the null model showed that about 2.1% (0.021) of the total variance was attributable to the community where the women live. Model III, which includes both individual and community-level variables, was chosen due to its low deviance (116,427). Therefore, Model III, the complete model with both the selected individual and household/community factors, was the best.

## Discussion

The pooled magnitude of unintended pregnancy in LMICs was 26.46%% (95% CI: 25.30%, 27.62%). The finding is higher than a study done in Six Asian countries (19.1%) (56) and lower than a study done in SSA (29.0%) (13). It is critical to recognize that, while unintended pregnancies are common in LMICS, some variations still exist across countries. The possible reason for this variation might be the difference in the health system of each country. According to our findings, unintended pregnancy rates in low- and middle-income countries range from 19.25% in Egypt to 61.71% in Bolivia.

In a multivariable multilevel logistic regression analysis, paternal education, working status, median exposure, household wealth index, residence, and distance to health facilities were significantly associated with unintended pregnancy in low and middle-income countries.

Women whose husbands have no education and primary education have higher odds of unintended pregnancy compared to women whose husbands have higher education (57, 58). Partners who have no formal education or a lower level of education are less likely to encourage their wives to use modern contraceptives and a woman's pregnancy intentions and parenting decisions are influenced by her partner's attitude (59). Improving the male partner's educational status is critical because the male partner has a strong influence on most household decisions, including the timing of pregnancy and the number of children desired (58).

Women who have media exposure have higher odds of unintended pregnancy compared to women who have no media exposure. This finding is contradictory to a study done in Ethiopia (44, 60), Nepal (61), and Pakistan (62). The possible justification might be even though media exposure creates awareness, that women having exposure to media may have an increased chance of social networks that may expose them to unintended pregnancy.

Women who are currently working have higher odds of unintended pregnancy compared to women who are not currently working. This finding is consistent with a study done in Ethiopia (63, 64) This finding is contradictory to a study done in Ethiopia (65) and Cambodia (66). The possible reason might be women with occupations may have a high level of social interaction and the nature of their work, which may lead to casual sex followed by unwanted pregnancy (63).

The likelihood of unintended pregnancy was higher among women who lived in a household with a male head than among women who lived in a household with a female head. The finding is similar to previous findings (67). Women living in male-headed households may not have the opportunity to actively participate in family planning decisions, resulting in an unmet need for family planning and unintended pregnancy (68).

Women with unmet family planning needs were more likely to experience unintended pregnancy than those with met needs. The finding is consistent with previous studies (69–73). The possible justification for this could be that the unmet need for contraception has exposed women to the risks of unplanned pregnancy. Addressing the challenges and unmet need for contraception should be a priority for reducing unintended pregnancies in low- and middle-income countries.

Women with a big problem with distance to health facilities have higher odds of having unintended pregnancies than women who do have not a big problem. The finding is consistent with a study done in Ethiopia (74–76). The possible reason might be that women facing a big problem of distance to health facilities may have a problem accessing health care such as contraception (77).

### Strengths and limitations of the study

The use of weighted, nationally representative large datasets of low and middle-income countries, with advanced statistical analysis techniques that account for the nature of DHS data for better parameter estimation was a strength of this study. However, due to the cross-sectional type of data, uncovering the causality between dependent and independent variables is challenging. The data was obtained through self-reports from women five years before the survey, which could be a cause of recall bias. Moreover, because it depends on the factors available in the DHS data set, the most significant explanatory factors such as medical-related factors, and the quality of maternal health services may be missed.

## Conclusion

Unintended pregnancy rates remain high in LMICs. Women whose husband has no education and primary education, women with media exposure, working status, women who live in a household headed by a male, women with unmet need for contraception, and women with a big problem of distance to health facilities were variables that were significant predictors of unintended pregnancy. When attempting to minimize unintended pregnancy in LMICs, these factors need to be considered. Furthermore, most of these attempts should be driven by government entities in low and middle-income countries.

## Data Availability

Publicly available datasets were analyzed in this study. This data can be found here: www.dhsprogram.com.
